# Retraction: Exploratory algorithms to devise multi-epitope subunit vaccine by examining HIV-1 envelope glycoprotein: An immunoinformatics and viroinformatics approach

**DOI:** 10.1371/journal.pone.0324076

**Published:** 2025-05-06

**Authors:** The PLOS One Editors

The *PLOS One* Editors retract this article [1] due to concerns about peer review integrity, authorship, and potential manipulation of the publication process. These concerns call into question the validity of the reported results. We regret that the issues were not identified prior to the article’s publication.

All authors did not agree with the retraction.
